# Reporting Incidents of Workplace Violence

**DOI:** 10.21315/mjms2024.31.6.18

**Published:** 2024-12-31

**Authors:** Yusrita Zolkefli, Nurul’Ain Ahayalimudin

**Affiliations:** 1 PAPRSB Institute of Health Sciences, Universiti Brunei Darussalam, Brunei Darussalam; 2 Department of Critical Care Nursing, Kulliyyah of Nursing, International Islamic University Malaysia, Malaysia

Dear Editor,

With great interest, we read the insightful paper entitled “A qualitative systematic review of healthcare practitioners’ experience of workplace violence” by Emary et al. (1). While this study has identified broader steps to protect the well-being of healthcare staff, for this commentary, we would like to focus on the reluctance of healthcare professionals to disclose the incident formally. The goal is to shed light on the implications of underreporting caused by such reluctance (2).

To begin with, Emary et al. (1) observed that some healthcare professionals seek comfort from peers or merely brush off the violent incident. This response may only provide a quick fix and is unlikely to be sustainable. We feel that there is an urgent need to report any form or degree of violence due to the greater harm it causes. Common violence, like verbal abuse, causes unnecessary emotions such as depression, anger, fear, and anxiety and may lead to long-term mental health issues (3). Because of this, underreporting would only mask the true nature and frequency of violent incidents, making them difficult to address. Low reporting also gives hospital officials a false sense of security, so they often fail to take precautions against violent incidents (4). Even worse, workplace violence will continue to be misinterpreted as inevitable, normal and acceptable.

As a way forward, we believe it is crucial to establish a voluntary reporting practice that begins with an open reporting culture. This can start with a practical incident reporting mechanism or tool that is user-friendly and quick to complete (5). This is followed by a meaningful reporting system, on which the report would result in positive impacts. Healthcare professionals have been sceptical and feel that reporting is worthless because “nothing comes of it” (6). Thus, it is insufficient to merely ask or expect healthcare professionals to report violent incidents voluntarily if doing so will not result in significant improvements.

In summary, Emary et al. (1) underscores the importance of safe and respectful workplaces for healthcare professionals. Thus, we advocate for sustainable and responsible reporting practices that uncover the real scope and frequency of workplace violence and help find better solutions to the pressing problem. We further believe reporting should be made as practical and meaningful as possible.
